# Assessment of Leptin Levels and Their Correlation With the Severity of Obstructive Sleep Apnea Syndrome: A Case-Control Study

**DOI:** 10.7759/cureus.42028

**Published:** 2023-07-17

**Authors:** Kuldeep Patial, Hara Prasad Mishra, Giridhari Pal, Tarun Kumar Suvvari, Chinmaya Mahapatra, Nidhal A Amanullah, Indrajeet Singh, SN Gaur, Rajendra K Behera

**Affiliations:** 1 Sleep Medicine Division, Vallabhbhai Patel Chest Institute, University of Delhi, New Delhi, IND; 2 Clinical Trial, All India Institute of Medical Sciences, New Delhi, IND; 3 Pharmacology and Therapeutics, University College of Medical Sciences, University of Delhi, New Delhi, IND; 4 Pharmacology, Vallabhbhai Patel Chest Institute, University of Delhi, New Delhi, IND; 5 Medicine and Surgery, Squad Medicine and Research (SMR), Visakhapatnam, IND; 6 Medicine and Surgery, Rangaraya Medical College, Kakinada, IND; 7 School of Pharmacy, The Neotia University, Sarisha, IND; 8 Psychiatry and Behavioral Sciences, Sree Ramakrishna Mission Hospital, Thiruvananthapuram, IND; 9 Biotechnology, Rama University, Kanpur, IND; 10 Pulmonary Medicine, Vallabhbhai Patel Chest Institute, University of Delhi, New Delhi, IND; 11 School of Life Sciences, Sambalpur University, Sambalpur, IND

**Keywords:** severity, osas, obesity, leptin, obstructive sleep apnea

## Abstract

Background

Obstructive sleep apnea (OSA) is characterized by a combination of structural issues in the upper airway and imbalances in the respiratory control system. While numerous studies have linked OSA with obesity, it remains uncertain whether leptin, a hormone associated with fat, plays a role in the functional and anatomical defects that lead to OSA. Therefore, the aim of this study was to investigate whether leptin levels could be used as a predictor of OSA syndrome (OSAS).

Methodology

A case-control observational study was conducted, enrolling study participants who reported obesity (BMI > 30) within the range of >30 to <35 kg/m^2^, along with a short neck and a history of snoring, excessive daytime drowsiness, fatigue, or insomnia. Leptin levels and fasting blood sugar (FBS) were measured in all individuals. Additionally, the study evaluated the severity of OSAS using indicators such as the STOP BANG scores, apnea-hypopnea index, uvula grade score, and Epworth Sleepiness Scale scores.

Results

A total of 80 participants (40 cases and 40 controls) were included in the study. The mean leptin and FBS levels were significantly higher in cases compared to controls. Moreover, leptin levels exhibited a significant correlation with the severity indices of OSAS.

Conclusion

The study findings indicate that individuals with higher leptin levels tend to exhibit more severe OSAS symptoms. Furthermore, these elevated leptin levels contribute to the worsening of various OSA symptoms. Larger controlled studies have suggested that pharmacologically restoring the altered leptin levels may serve as a beneficial adjunct to treatment for alleviating OSAS symptoms.

## Introduction

Obstructive sleep apnea (OSA) is a condition that arises from a combination of physiological changes in the upper airway and abnormalities in the respiratory control system. It should not be considered solely as a local respiratory disorder, as it has systemic consequences. OSA affects individuals of all ages and is relatively common in the general population [1]. The worldwide prevalence of OSA is 54%, and its prevalence in North America, Europe, and China ranges from 9% to 38% and 8.8% to 24.2% [2]. Previous studies have shown a steady increase in the number of OSA syndrome (OSAS) patients. In North India, the prevalence of OSAS is comparable to other serious conditions such as diabetes mellitus, obesity, and hyperuricemia, occurring in about 13.7% of habitual snorers and 3.6% of non-habitual snorers (with a prevalence of 4.9% in men and 2.1% in women) [2,3]. Untreated OSAS patients have been reported to be at risk of accidents due to excessive daytime sleepiness, psychomotor deficits, decreased productivity, and absenteeism [3-6]. While the majority of obese individuals have elevated levels of circulating leptin, the pathophysiological mechanisms underlying the etiology of OSA remain unclear.

Leptin, a 167-amino-acid protein primarily produced by adipocytes, is encoded by the obesity gene and has a molecular weight of approximately 16 kDa. It is expressed in various tissues, including the mammary gland, placenta, and stomach. Leptin levels are closely associated with fat mass and play a role in regulating appetite, energy expenditure, and lipid and carbohydrate metabolism through hypothalamic processes [7]. Serum leptin levels increase with obesity and are strongly correlated with fat mass and body mass index (BMI) [7]. In obese individuals, hyperleptinemia occurs due to an abundance of unbound leptin, whereas lean individuals primarily have the bound form of leptin [7]. This suggests that obesity is often characterized by leptin resistance rather than a defect in the obesity gene itself. The hypothesis that leptin deficiency is related to apnea has been proposed based on the finding that leptin acts as an appetite suppressant and ventilatory stimulant in rats, as well as the strong association between obesity and OSAS in humans. Recent studies related to metabolic syndrome have shown that individuals with OSAS have higher levels of leptin, indicating the presence of relative leptin resistance and insulin resistance [8]. There may be a connection between OSA and metabolic syndrome; therefore, the threshold for establishing OSA may need to take metabolic syndrome into account. Further investigations, including genetic analysis and the study of adipokines, may be necessary to better understand the relationship between OSA and metabolic syndrome. The polymorphism of the leptin receptor gene has been implicated in OSA in multiple instances [9,10].

Although several studies have been conducted, there is still no definitive evidence linking OSA and metabolic syndrome. However, intermittent hypoxia resulting from OSA can accelerate the development of metabolic syndrome, and the metabolic syndrome itself can contribute to the functional and anatomical changes that lead to OSA. As a result, it remains unclear whether OSA causes metabolic syndrome or if there are common underlying factors contributing to both conditions. The aim of this case-control study is to assess the potential of leptin as a predictor for OSA to correlate with the severity of OSAS.

## Materials and methods

Study center

The current study was carried out in two centers: Department of Life Sciences, Sambalpur University, Sambalpur, India, and Sleep Laboratory, Department of Pulmonary Medicine, Vallabhbhai Patel Chest Institute, University of Delhi, New Delhi, India. The study period was two years.

Subject recruitment

The study participants were adult males aged 30-60 years seeking medical assistance for snoring, daytime drowsiness, or both at the hospital’s outpatient department (OPD). The specific age group was selected because age-related sleep problems are more common in this age range. A total of 80 participants were recruited (40 controls and 40 OSA patients). Individuals aged 30-60 years were assessed for obesity (defined as having a BMI greater than 30) within the range of greater than 30 kg/m^2^ to less than 35 kg/m^2^, as well as other factors such as a short neck, a history of snoring, excessive daytime drowsiness, fatigue, or insomnia. Before being enrolled in the study, each participant was provided with written details regarding the research in the form of a patient information sheet. A patient permission form was used to acquire their informed consent after they got a thorough explanation of the study. Prior to recruiting participants, the institute’s human ethics committee gave its approval to the study protocol.

Sample size

With one control for every instance, we conducted an independent case-control study. Previous research has found that the likelihood of exposure for controls is 13%. We must examine at least 40 occurrences, and control patients must reject the null hypothesis that this odds ratio is equal to one with a probability (power) of 0.8 if the true odds ratio for illness for exposed vs. unexposed individuals is 5. The null hypothesis test’s type I error probability was calculated using a significance level of 0.05. Either Fisher's exact test or the continuity-corrected chi-squared statistic was used to test the null hypothesis. The sample size calculation was performed using PS version 3.1.2.

Study groups

A case group of 40 patients with newly identified untreated obesity-related OSAS were included in the study. BMI larger than 30 kg/m^2^, apnea-hypopnea index (AHI) more than 5 per hour, and Epworth Sleepiness Scale (ESS) score equal to or greater than 10 points were all present in the case group patients. A control group of 40 patients were included. Using the proper statistical techniques, additional age-matched, healthy controls from group B were compared to the patients in group A. Routine tests were used to examine the following variables in control and OSAS patient groups. The same observer conducted each measurement. All individuals had a thorough clinical evaluation. The recruited patients underwent the following biological tests using fasting venous samples, such as leptin level and fasting blood sugar (FBS). 

Sample collection and processing

After overnight polysomnography (PSG) recording and overnight fasting of at least 12 hours for untreated patients, 5 mL of venous blood was aseptically collected from the patient and placed in an EDTA vial between 7 and 8 am [11], and the samples were processed in accordance with the method of Seicean et al. [12]. In summary, the samples were subjected to centrifugation at 3,000 rpm for a duration of 20 minutes at a temperature of 0°C. After centrifugation, serum aliquots were isolated and stored at −80°C for subsequent biochemical analysis [12,13].

Assessment of leptin level and fasting blood glucose level

Using a readily accessible ELISA kit and adhering to the manufacturer’s instructions, leptin levels were measured [14]. According to the manufacturer’s recommendations, FBS levels were tested using an autoanalyzer (measured after an eight-hour or overnight fast) [15]. Using venous blood samples taken in the morning, the concentration of glucose in the serum was determined. FBS was analyzed to determine the glucose level, and the hexokinase G-6-PDH technique was utilized. Subsequently, the blood sample was subjected to centrifugation.

Assessment of OSA measurement parameters

According to Mbata and Chukwuka, AHI can be used to diagnose OSA [16]. Additionally, AHI can be used to detect apnea among study participants [16]. The number of apnea or hypopnea occurrences that occur during a night is multiplied by the number of hours of sleep to get the AHI, and BP has no direct correlation. The classification of sleep apnea severity is as follows: normal sleep (AHI averaging fewer than five events per hour), mild sleep apnea (AHI ranging between five and 14 incidents per hour), moderate sleep apnea (AHI between 15 and 29 incidences per hour), and severe sleep apnea (AHI of 30 or more events per hour). 

The AHI is calculated by dividing the total number of events (apneas and hypopneas) by the total sleep time. According to AASM criteria, if an incident lasts at least 10 seconds, is accompanied by a drop in blood oxygen levels, or causes an awakening from sleep, it is categorized as apnea or hypopnea. These sleep disruptions caused by OSA can reduce restfulness and contribute to daytime fatigue.

Assessment of ESS 

Johns developed the ESS in 1991 to offer a purely subjective indicator of sleepiness during the day [17]. It is commonly utilized as a sleepiness indicator for conditions like OSA and other disorders, and its validity has been confirmed through translations and validations in multiple languages. A score equal to or greater than 10 on the scale is often indicative of sleepiness.

Assessment of STOP BANG 

Participants were required to complete a questionnaire with questions on the STOP BANG and ESS after the informed consent procedure was complete. Individuals with a STOP BANG score of 3 or higher were notified and provided with instructions to seek further medical attention if deemed necessary. Descriptive statistics were employed to analyze demographic data, while the relationship between risk variables and the STOP BANG score was assessed using the chi-square test with Yates’ correction [18].

Assessment of uvula grade

The patient is examined while seated, their head held neutrally, their mouth open, and their tongue stretched as far as it will go without producing any discomfort. The visibility of the airway structures is used to determine the grade. Grade I indicates that the pillars, tonsils, and soft palate are all visible. Grade II indicates the visibility of the pillars, uvula, and upper pole. Grade III signifies visibility of the pillars, hard palate, and upper pole. Grade IV indicates that only the hard palate is visible. There is generally a high level of agreement among observers when categorizing patients based on these criteria [19].

Ethical consent

The “Declaration of Helsinki” and the Central Ethics Committee on Human Research’s (CECHR) ICMR-2000 guidelines for ethical conduct in biomedical research on humans were both adhered to in this project. Only after completing a consent form were patients and controls allowed to participate in the intended study.

Statistical analysis

The data were presented as mean values with the standard error of the mean (SEM) included. IBM SPSS Statistics, version 20.0 (IBM Corp., Armonk, NY) was used for statistical analysis. The Pearson chi-square test was used to evaluate categorical data, while the student t-test was used to evaluate quantitative data. The associations between outcome parameters were investigated using the Pearson coefficient of correlation test. The cutoff for statistical significance in each test was set at a significance level less than 0.05 (p ˂ 0.05).

## Results

**Figure 1:**
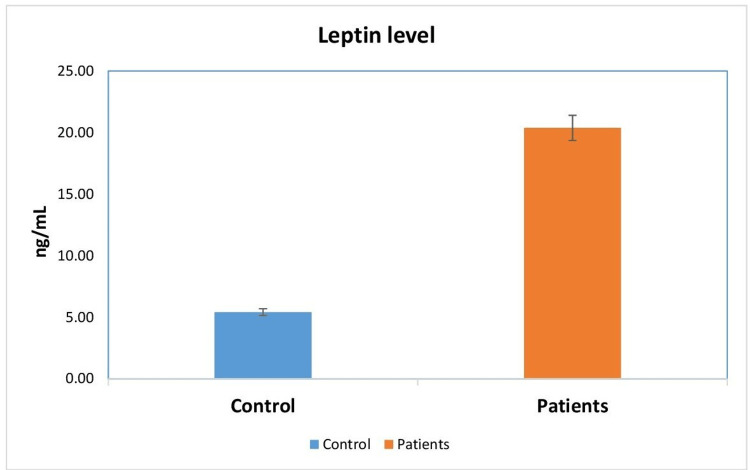
The distribution of individuals in the experimental and control groups based on their mean leptin levels

**Figure 2:**
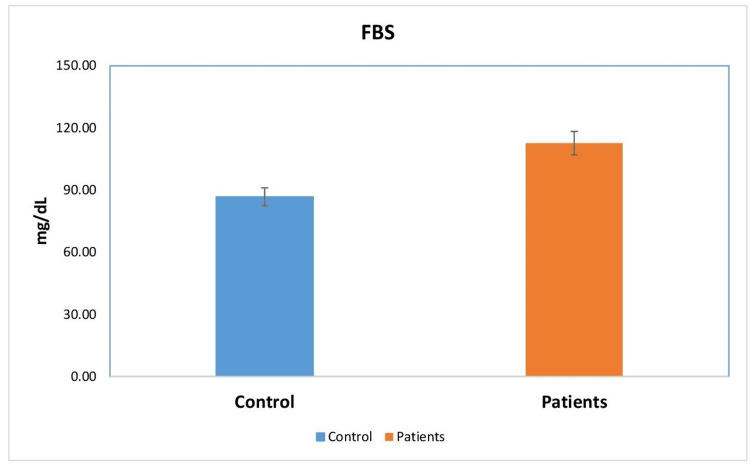
The distribution of individuals in the experimental and control groups based on their mean FBS levels FBS, fasting blood sugar

A total of 80 participants (40 cases and 40 controls) were included in the study. The mean leptin levels in the patient group were significantly higher compared to those in the control group (22.57 ± 4.23 vs. 5.71 ± 3.02; p < 0.01). This suggests that the average leptin level in the patient group was 22.57 ng/mL, whereas the control group had an average leptin level of 5.71 ng/mL. The distribution of individuals in the experimental and control groups based on their mean leptin levels is depicted in Figure 1. Additionally, the mean FBS levels in the patient group were significantly higher compared to those in the control group (113.14 ± 8.72 vs. 87.29 ± 13.26; p < 0.01). This implies that individuals in the control group had an average FBS level of 87.29 mg/dL, while those in the patient group had an average FBS level of 113.14 mg/dL. Figure 2 depicts the distribution of individuals in the experimental and control groups based on their mean FBS levels.

**Figure 3:**
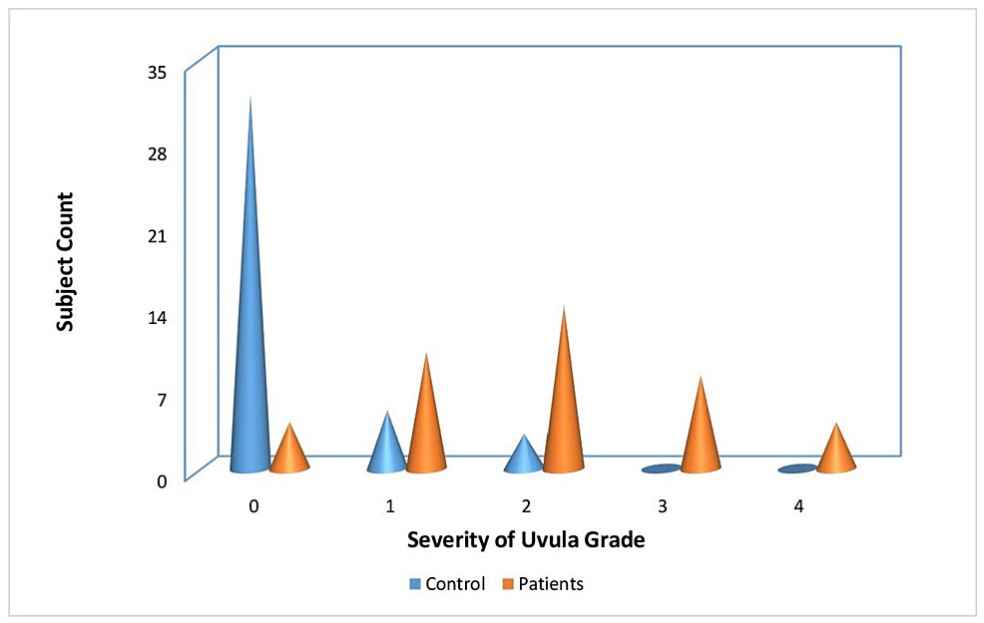
The distribution of study participants in the control and experimental groups based on the severity of uvula grade

**Figure 4:**
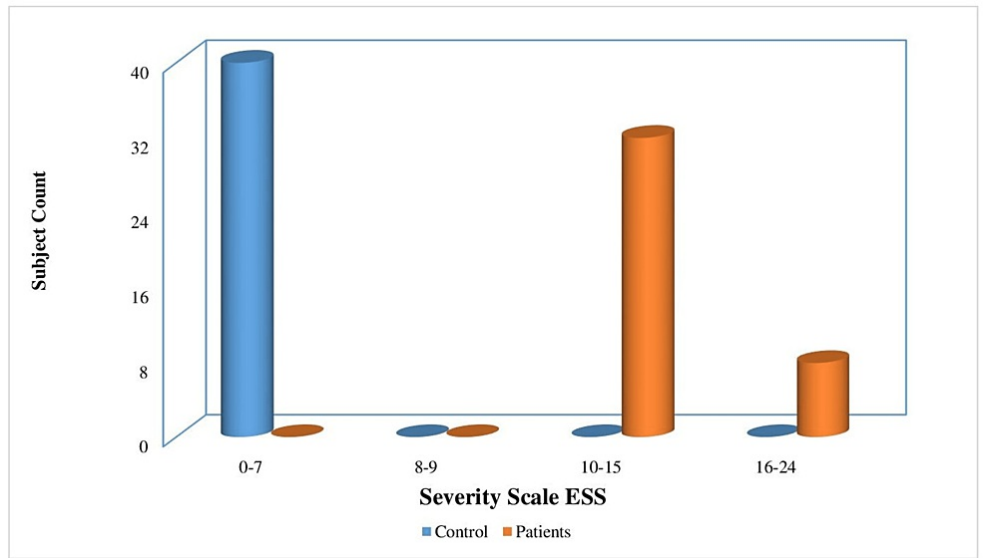
The distribution of study participants in the control and experimental groups based on the severity of ESS ESS, Epworth Sleepiness Scale

In the control group, 80% of the participants had uvula grade scores within the normal range, while 12.5%, 7.5%, and 40% of them had mild to moderate uvula grade scores. Therefore, it can be inferred that individuals in the patient group had abnormal uvula grade scores, whereas those in the control group had normal uvula grade scores. The distribution of study participants in the control and experimental groups based on the severity of uvula grade is depicted in Figure 3. Regarding the ESS scores, 80% of the patients in the experimental group displayed moderate ESS scores, while 20% had severe ESS scores. In contrast, the control group had ESS scores within the normal range. Hence, it can be inferred that participants in the control group had normal ESS scores, whereas those in the patient group had abnormal ESS scores. The distribution of study participants in the control and experimental groups based on the severity of ESS is depicted in Figure 4.

**Figure 5:**
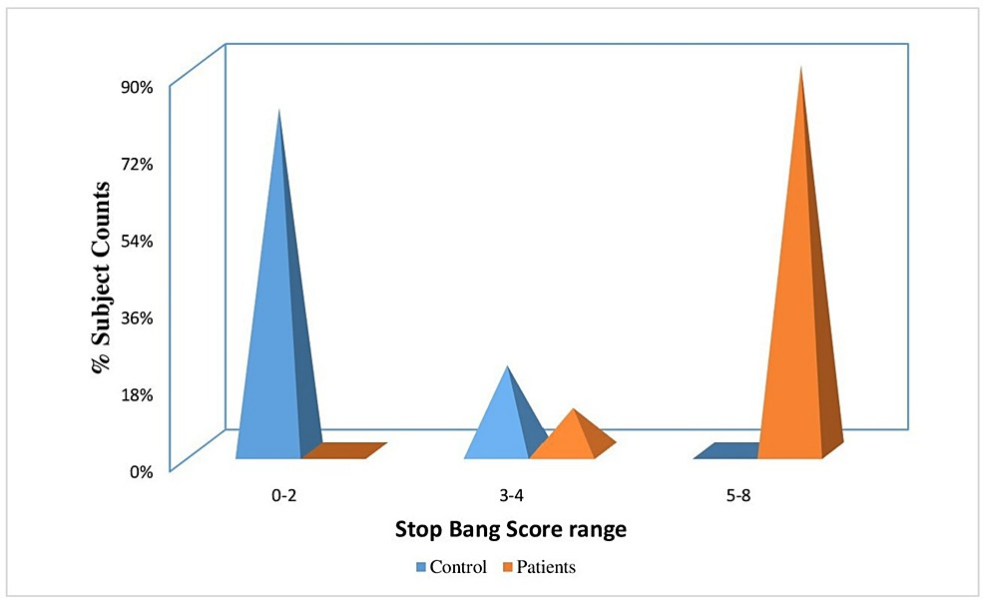
Percentage distribution of individuals in the control and experimental groups based on the severity of STOP BANG scores

**Figure 6:**
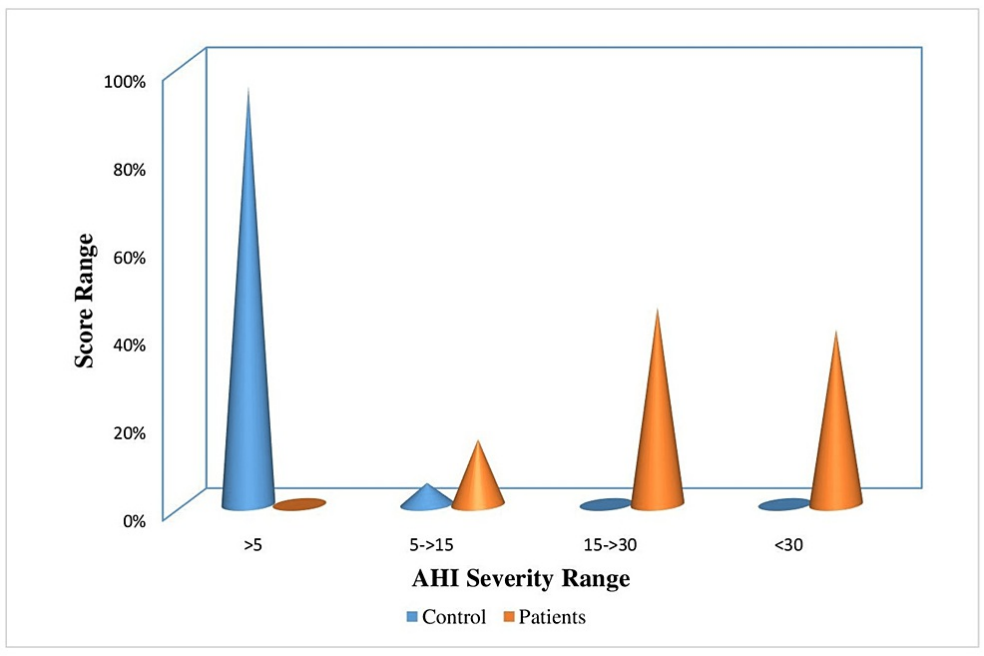
Percentage distribution of individuals in the control and experimental groups based on the AHI AHI, apnea-hypopnea index

Figure 5 depicts the distribution of individuals in the control and experimental groups based on the severity of STOP BANG scores. In the control group, 80% of individuals fell into the normal class, while 20% belonged to the mild class. On the other hand, in the patient group, 90% of individuals had moderate STOP BANG scores and 10% had severe scores. Therefore, it can be inferred that the patient group had abnormal STOP BANG scores, while the control group had STOP BANG scores within the normal range. Figure 6 depicts the distribution of individuals in the control and experimental groups based on the AHI. In the control group, 95% of individuals fell into the normal category, while 5% were categorized as mild. In contrast, among the patient group, 15%, 45%, and 40% of individuals had mild, moderate, and severe AHI score ranges, respectively. Thus, it can be deduced that the patient group exhibited abnormal AHI scores, whereas the control group had AHI scores within the normal range.

The correlation between leptin and the various OSAS parameters is described in Table 1. The results indicate a significant correlation between the severity of OSAS and uvula grade, AHI score, ESS score, and STOP BANG score. These findings suggest a strong association between these variables and the severity of OSAS in both groups.

**Table 1:** The correlation between leptin and the various OSAS parameters OSAS, obstructive sleep apnea syndrome; AHI, apnea-hypopnea index; ESS, Epworth Sleepiness Scale

Variables	Chi-value	p-value
Uvula grade and severity of OSAS	21.47	<0.001
AHI score and severity of OSAS	38.45	<0.001
ESS score and severity of OSAS	47.23	<0.001
STOP BANG and severity of OSAS	26.25	<0.001

## Discussion

OSA is a well-recognized medical condition with significant implications for public health, leading to long-term negative health effects such as metabolic, vascular, and cardiac diseases [20,21]. Lifestyle factors play a significant role in the development of OSAS compared to structural causes [20]. Moreover, these lifestyle factors can contribute to the development of leptin resistance [21]. The narrowing of the upper airway due to anatomical and physiological factors, including obesity and structural changes, is a key factor affecting its patency. In addition, reduced neuromuscular compensation and the absence of the pharyngeal protective reflex during sleep contribute to increased collapsibility of the pharynx. The exact pathophysiological processes underlying OSA development are not fully understood, but it has been observed that obese and diabetic individuals often exhibit abnormal levels of leptin and FBS, which may be indicative of OSA-related issues [22].

In our study, there was a significant difference in leptin levels between the control group and the group with OSAS. However, previous research did not find a noticeable distinction in leptin levels between individuals with OSAS and control subjects [2,3,9]. Nevertheless, several studies have demonstrated that individuals with OSA tend to have higher leptin levels compared to non-apneic individuals with similar weight and BMI [2,3,9]. This suggests that OSA leads to both leptin resistance and adiponectin resistance. Our findings support a recent study by Ursavas et al., which also reported elevated leptin levels in individuals with OSAS, indicating the presence of leptin resistance [23]. The AHI is used to classify the severity of OSA as mild to moderate (5 < AHI < 30) or severe (AHI ≥ 30). In our study, a higher proportion of patients in the patient group had elevated AHI levels compared to the control group, indicating a more severe case of OSAS. A previous study conducted by Arnardottir et al. analyzed various studies that investigated the prevalence of OSA in the general population using an AHI threshold of 15 or above [24]. The AHI was used as a metric in the study to further explore the relationships between OSA, fatigue, sleep-related symptoms, and attentiveness.

The ESS is not a reliable predictor of OSA, but when elevated, it can be predictive of a positive response to therapy. In our study, 20% of the patients had moderate ESS scores, while 80% had severe scores. Previous research has shown that higher ESS scores are associated with increased daytime sleepiness. A person with an ESS score of 10 or higher is considered sleepy. The STOP BANG model score proved to be useful for categorizing individuals with sleep apnea. We chose the STOP BANG paradigm for its simplicity and practicality. Some studies have suggested a correlation between the severity of OSA, as indicated by the AHI, and the score obtained from the STOP BANG model. Improved analysis using relative odds logistic regression can be applied to classify ordinal data. In our study, a larger proportion of participants in the patient group had higher uvula grade scores compared to the control group, indicating a more severe case of OSAS. Previous studies have established a correlation between uvula size, snoring, and OSA, with larger uvulas often associated with OSA and loud snoring [25]. The AHI index, ESS score, STOP BANG score, and uvula grade scores were all indicators of the severity of OSAS in our study. These indicators were found to be correlated with plasma leptin levels. There were significant differences in leptin levels and severity criteria for OSAS between the patient population and the control group. These findings align with a previous investigation by Ozturk et al., who found a positive correlation between plasma leptin levels and the degree of sleep-disordered breathing measured by OSAS indices [26,27].

The limitations of our study are small sample size and recall bias as we relied on self-reported measures, such as the ESS. Moreover, we haven’t considered potential confounding factors that are important such as comorbidities or medication use, which could influence the associations between the studied variables.

## Conclusions

Our study findings indicate a correlation between higher leptin levels and the severity of OSAS. Moreover, elevated leptin levels may contribute to worsening symptoms of OSA. Promisingly, larger controlled studies have suggested that pharmacological interventions aimed at modulating leptin levels may offer a promising adjunctive treatment approach, potentially alleviating symptoms associated with OSAS. However, future research should also explore the potential benefits of comprehensive treatment strategies that incorporate lifestyle management, addressing factors such as weight loss, exercise, and sleep hygiene. Integrating these approaches may provide a more holistic and effective approach to managing OSAS and improving patient outcomes.
